# Influence of the Heat Treatment on the Particles Size and on the Crystalline Phase of TiO_2_ Synthesized by the Sol-Gel Method

**DOI:** 10.3390/ma11122364

**Published:** 2018-11-24

**Authors:** Michelina Catauro, Elisabetta Tranquillo, Giovanni Dal Poggetto, Mauro Pasquali, Alessandro Dell’Era, Stefano Vecchio Ciprioti

**Affiliations:** 1 Department of Engineering, University of Campania “Luigi Vanvitelli”, Via Roma 29, I-81031 Aversa, Italy; elisabetta.tranquillo@unicampania.it; 2 Department of Biochemistry, Biophysics and General Pathology, University of Campania “Luigi Vanvitelli”, Medical School, Via L. De Crecchio 7, 80138 Naples, Italy; 3 ECORICERCHE, Srl, Via Principi Normanni, 81043 Capua (CE), Italy; giogiodp@hotmail.it; 4 Department S.B.A.I., Sapienza University of Rome, Via del Castro Laurenziano 7, I-00161 Rome, Italy; mauro.pasquali@uniroma1.it (M.P.); alessandro.dellera@uniroma1.it (A.D.); stefano.vecchio@uniroma1.it (S.V.C.)

**Keywords:** sol-gel method, FTIR, nanoparticles size, titania, anatase, rutile

## Abstract

Titanium biomaterials’ response has been recognized to be affected by particles size, crystal structure, and surface properties. Chemical and structural properties of these nanoparticle materials are important, but their size is the key aspect. The aim of this study is the synthesis of TiO_2_ nanoparticles by the sol-gel method, which is an ideal technique to prepare nanomaterials at low temperature. The heat treatment can affect the structure of the final product and consequently its biological properties. For this reason, the chemical structure of the TiO_2_ nanoparticles synthesized was investigated after each heat treatment, in order to evaluate the presence of different phases formed among the nanoparticles. FTIR spectroscopy and XRD have been used to evaluate the different structures. The results of these analyses suggest that an increase of the calcination temperature induces the formation of mixed-crystalline-phases with different content of anatase and rutile phases. The results obtained by SEM measurements suggest that an increase in the particles size accompanied by a noticeable aggregation of TiO_2_ nanoparticles is due to high temperatures achieved during the thermal treatments and confirmed the presence of different content of the two crystalline phases of titanium dioxide.

## 1. Introduction

Nanotechnology is the science and the engineering involved in the design, syntheses, and characterization of materials whose particles size is on a nanometer scale. Nanomaterials have different chemical properties compared to bulk materials, in fact they were studied since their specific functions depend of their shape and size [1].

In the last decade, different nanostructured oxide materials such as ZrO_2_, SiO_2_, and TiO_2_ or ZnO have been developed in many fields [2,3,4,5,6]. In particular, titanium dioxide (TiO_2_) is frequently used in industrial and environmental applications for its photocatalytic activity, chemical stability, and anticorrosive properties. The good biocompatibility of this nanomaterials makes them excellent candidates for biomedical applications.

It has been reported in literature that titanium biomaterials’ response is affected by their size, crystal structure, and surface properties [7]. Chemical and structural properties of these nanoparticle materials are important, but their size is the key aspect. Titanium particles larger than 100 nm, with particular reference to those located in the bulk, have been considered biologically inert in humans bodies [8]. Therefore, to improve the biological properties of this titania nanoparticles their surface and structure have been modified by coating or by surface functionalization [9,10]. The high surface area of nanoparticles with lower size (<100 nm) enhances the cellular adhesion promoting the interaction between nanoparticles and tissues or cells, thus allowing penetration of the nanoparticles through the cell membrane [7,11]. Actually, titania nanoparticles are used for several biomedical applications, such as drug delivery, markers for bioimaging, magnetic resonance imaging (MRI), tissue repair [12], as well as in cancer therapy [13].

The synthesis technique widely affects several properties of the TiO_2_ nanoparticles. Many procedures to synthetize these nanoparticles are reported in literature such ultrasonic spray pyrolysis method [14], semi-batch/batch two stage mixed method [15], microemulsion method [16], and the sol-gel process [17]. Among these synthesis technique, the sol-gel method is the most suitable technique to prepare them at low temperature [18]. The advantages of this method are its versatility, the high degree of purity of the products and the possibility to obtain a fine control of the microstructure of the final product by modulation of the synthesis parameters [19,20]. Formation of TiO_2_ nanoparticles is due to the occurrence of hydrolysis and condensation reactions on the metal alkoxide precursor. In this synthesis process, the suitable choices of metal alkoxide precursor and reactions conditions play an important role on the size of the resulted nanoparticles [21,22]. The concentration of precursor could determine the different formation of primary particles that occurs through the nucleation process. Aggregation of the primarily formed particles during the condensation reactions leads to the formations of more stable particles with a larger size. However, it is possible to slow down the crystal growth with the view to obtain smaller particles size by tuning the operative conditions of the synthesis (i.e., reducing the preparation rate) [23].

The heat treatment can affect the structure of the final product and consequently its biological properties [24]. High temperatures can induce the formation of different crystalline phases with different characteristics. In fact, Chan Jin et al. [25] reported that the different titania phase exhibit several biological activity. TiO_2_ NPs in the anatase phase cause cytotoxicity but also genetic toxicity, because they are internalized in cytoplasm and some of them are lodged inside the mitochondria and in nucleus. The authors concluded that TiO_2_ NPs in the anatase phase cause ultrastructural damage of cells though the generation of Reactive Oxygen Species (ROS) and have much more toxicity than the other crystal phases. These effects could depend by the different crystal structure and size. Titania has three mineral forms: anatase, rutile and brookite. Anatase and rutile have a tetragonal crystal structure, and were widely studied for their different photocatalytic activity [26], while, instead, brookite has an orthorhombic crystalline structure [26].

The aim of this work is the synthesis of the TiO_2_ nanoparticles by sol-gel method and, in particular, how the heat treatment affects the TiO_2_ nanoparticles crystal phases and their size. Different parameters such as chemical and crystal structure have been studied by FT-IR spectroscopy and XRD. Furthermore, the morphology and nanoparticles size of the prepared TiO_2_ nanoparticles have been investigated by SEM.

It is well-known that the crystal structure can influence both the nanoparticles size and the biological properties [8,25,27,28,29,30,31]. So, for this reason the chemical study is of crucial importance, and it is focused on investigating the direct interaction between the nanoparticles and the cells, with the view to use these materials in the biomedical field, such as drug delivery system or markers for bioimaging.

## 2. Materials and Methods

### 2.1. Reagents

The chemicals used in this study were titanium (IV) butoxide (TBT, Sigma Aldrich, St. Louis, MO, USA), pure ethanol (99.8% Sigma-Aldrich) and distilled water, used as received.

### 2.2. Sol–Gel Synthesis

**Figure 1:**
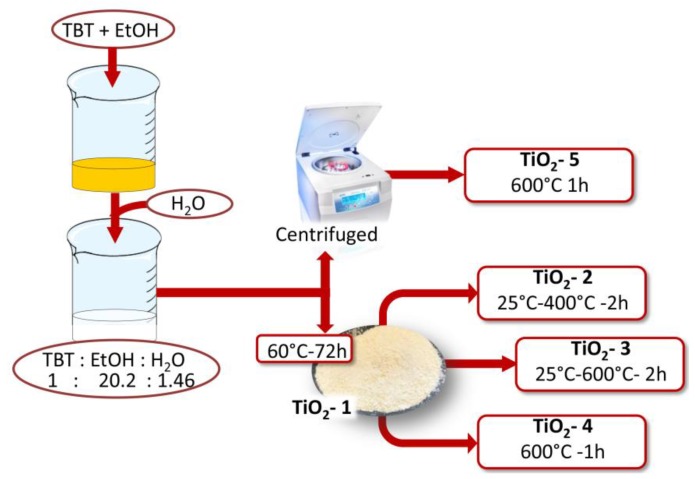
Flow chart of TiO_2_ nanoparticles by the sol-gel method and the molar ratios between the reagents achieved in the sol.

The TiO_2_ nanoparticles were obtained by the sol-gel method through hydrolysis and condensation reactions of the precursor. Titanium (IV) butoxide, the metal alkoxide precursor, was added in a solution of pure ethanol and distilled water (Figure 1).

Ethanol was used to dissolve TBT, and water because it allows its hydrolysis. The solution containing TBT and ethanol was subjected to magnetic stirring for 15 min, and finally distilled water was added drop by drop in the resulted solution according to the following molar ratios: TBT:EtOH:H_2_O = 1:20.2:1.5. Once the dripping process was completed, precipitation of the nanoparticles was observed. To determine the effect of the heat treatments on the size and properties of TiO_2_ nanoparticles, the sol was separated in two parts, and they were treated in different methods (Table 1). The former was placed in an oven for 72 h at 60 °C (TiO_2_-1), and the second was centrifuged, after three washings with ethanol and water, the supernatant was removed and the wet precipitates were placed in a hot muffle at 600 °C and left stable for 1 h (TiO_2_-5).

After the thermal treatment at 60 °C, TiO_2_-1 was divided into three aliquots and each of them was treated in muffle at different temperatures. The first and the second thermal treatments were carried out at 400 °C (TiO_2_-2) and 600 °C (TiO_2_-3), respectively. The temperature was increased from room temperature up to 400 °C and 600 °C at a rate of 9 °C/min. The samples were left stable for 2 h at these temperatures. The last sample was placed in the hot muffle at 600 °C for 1 h (TiO_2_-4).

### 2.3. Characterization

#### 2.3.1. Fourier Transform Infrared Spectroscopy (FTIR)

The chemical structure of the TiO_2_ nanoparticles was investigated after each heat treatment in order to evaluate the presence of different phases formed among the nanoparticles.

The synthesized nanomaterials were analyzed using a Prestige 21 Shimadzu Fourier transform infrared (FTIR) spectrophotometer (Tokyo, Japan) by collecting transmittance spectra in the 400–4000 cm^−1^ region with a resolution of 4 cm^−1^ (45 scans). The instrument was equipped with a DTGS KBr (Deuterated Tryglycine Sulphate with potassium bromide windows) detector. Pelleted disks containing 2 mg of sample diluted with KBr (sample to KBr ratio = 1:100) were made. FTIR spectra were finally analyzed by Prestige software (IR solution, 1.50, Shimadzu, Kyoto, Japan).

#### 2.3.2. Scanning Electron Microscopy (SEM) and X-ray Diffraction (XRD)

The morphology and nanoparticles size of TiO_2_ were investigated by scanning electron microscopy analysis performed using an AURIGA Zeiss High Resolution Field Emission equipment (HR-FESEM, model AURIGA; Carl Zeiss Microscopy GmbH, Jena, Germany).

Before the SEM analysis, the ethanol suspensions of the nanoparticles were prepared. Subsequently, they were centrifuged for few minutes to obtain nanoparticles solutions with low agglomeration. Afterwards, clean glass capillaries were used to transfer a droplet of each sample of suspension on the stub of the SEM. The ethanol was used as solvent for the suspension for its high rate of the evaporation process, in order to avoid the agglomeration of nanoparticles during the SEM analysis. In fact, the drying of the samples suspension on the stub of the SEM was occurs very quickly and then micrographs were taken at a number of random locations on the grid.

The crystalline phases have been identified by XRD analysis using a Philips diffractometer (Philips, Amsterdam, The Netherlands) equipped with a PW 1830 generator, tungsten lamp and Cu anode, where the source of X-ray is given by a Cu-Kα radiation (λ = 0.15418 nm).

## 3. Results

### 3.1. Nanoparticle Synthesis

**Figure 2:**
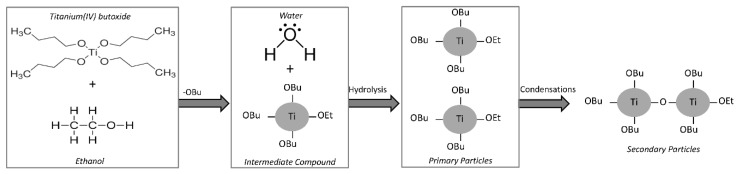
TiO_2_ nanoparticle formation mechanism.

The mechanism of the nanoparticles formation by hydrolysis and condensations reactions starting from addiction of water to the solution of TBT in ethanol is depicted in Figure 2. Precipitation of nanoparticles leads to a change of the color solution from colorless to opalescent white. The fast initial hydrolysis stage of the alkoxide precursor leads to the formation of primary particles. Aggregation of the primary particles is due to condensation reaction, which causes the formation of stable secondary particles with larger size [32,33]. At first, as it is possible to see in Figure 2, the OR groups of the secondary particles are the residue of the solvents used in the sol-gel synthesis, but, when the hydrolysis is almost complete all the organic (R) groups are removed and replaced by OH groups.

The concentration of TBT is an important parameter of synthesis. Indeed, a high amount of TBT, induces the production of a greater number of primary particles, which aggregate and grow until all the primary particles are consumed. Furthermore, the size of nanoparticles is also influenced by water concentration—a high concentration of water induces a high nucleation rate. Therefore, a greater amount of particles were produced and their agglomeration causes the formations of larger particles [23].

Moreover, it is well-known [34] that if water is added to ethanol before the addition of TBT, the formation of strong hydrogen bonds between water and ethanol hinders TBT hydrolysis. On the other hand, TBT is immiscible in water. For this reason, during the synthesis process TBT was previously dissolved in ethanol, and subsequently water was added to the resulted TBT–ethanol solution. When TBT has been added to ethanol, the formation of intermediate compounds is induced by the high reactivity of the alkoxide, favoring the exchange with the alcohol molecules, which contain active hydrogen atoms [17], as shown in Figure 2.

### 3.2. Chemical Characterization

#### 3.2.1. XRD Analysis

**Figure 3:**
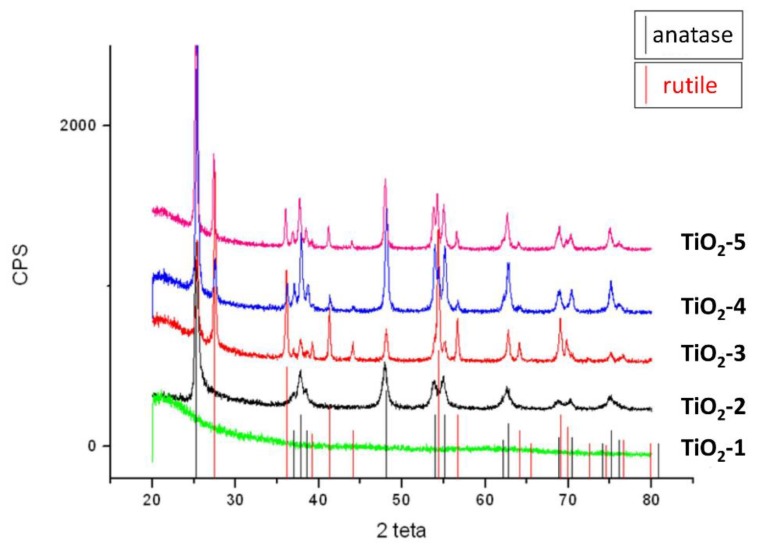
XRD of the TiO_2_-1 treated at 60 °C-72 h; TiO_2_-2 treated from 25 °C to 400 °C-2 h at 9 °C/min; TiO_2_-3 treated from 25 °C to 600 °C-2 h at 9 °C/min; TiO_2_-4 treated at 600 °C-1 h; TiO_2_-5 wet precipitates treated at 600 °C-1 h.

The formation of crystal phases of TiO_2_ nanoparticles was analyzed by XRD measurements. The TiO_2_-1 nanoparticles were amorphous, as it is clearly observed in Figure 3 by the absence of detectable peaks in the XRD spectrum. Increasing the temperature, the partials crystallization occurred in the samples from TiO_2_-2 to TiO_2_-5. Anatase was the only crystalline phase detected in TiO_2_-2 heated at 400 °C, while a mixture of both anatase and rutile phases (in different amount for each sample) was found in the other samples heated at 600 °C.

The occurrence of phase transformations is influenced by the temperature of the thermal treatment and surface defects of the materials [35,36]. The primary particles formed by the initial hydrolysis reactions usually contain many defects sites, which are due to dehydroxylation. This process leads to formation of oxygen vacancies by oxygen surface desorption [37,38], and when the materials were heated up to 400 °C, an atoms rearrangement and the phase transformation from anatase to rutile occur [39]. The XRD diffraction patterns of the samples (TiO_2_-3, TiO_2_-4, and TiO_2_-5) treated at 600 °C show in Figure 3 that the materials became a mixture of crystalline phases at different percentages of anatase and rutile, whose amount have been reported in Table 2, respectively. These results suggest that anatase is not completely transformed into the rutile phase at 600 °C.

**Figure 4:**
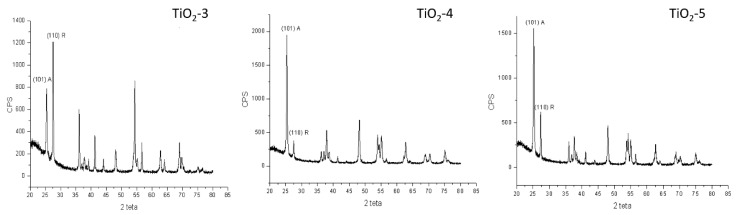
XRD patterns for samples TiO_2_-3, TiO_2_-4, and TiO_2_-5 with the main peak for both phases.

In Figure 4, there are reported only the spectra of the materials that contain both phases in order to analyze the intensity of the main peaks for each phase indexed and to evaluate the content of both phases.

The content of both anatase and rutile phases for all the TiO_2_ samples were calculated according to the following equation [40]:

X_A_ = 100/(1 + 1.265 IR/IA)

and the values so obtained have been reported in Table 2, where X_A_ is the weight fraction of anatase in the mixture, IA and IR are the intensities of the (101) anatase and (110) rutile diffraction peaks, respectively, estimated from the XRD spectra in Figure 4.

#### 3.2.2. FTIR Analysis

**Figure 5:**
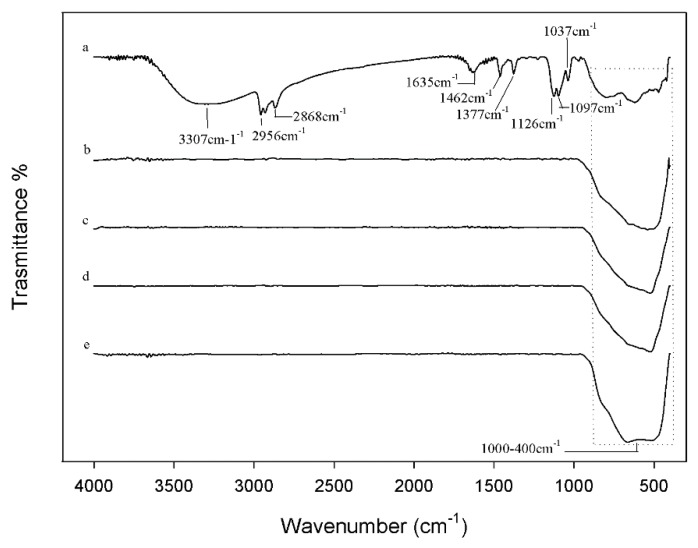
FTIR of (**a**) TiO_2_-1; (**b**) TiO_2_-2; (**c**) TiO_2_-3; (**d**) TiO_2_-4; (**e**) TiO_2_-5.

Figure 5 shows the FTIR spectra of all the synthesized nanoparticles after heating at different temperatures initially characterized by XRD. In the spectrum of TiO_2_ after the thermal treatment at 60 °C (curve a) the bands related to the stretching of -CH_2_ and -CH_3_ groups of the TBT precursor were observed at about 1400 cm^−1^, along with those at 2956 and 2868 cm^−1^ due to the asymmetric and symmetric stretching vibrations of the methyl groups, respectively [21,41]. The typical bands of the ethanol attributed to the C–O group at 1126, 1097, and 1037 cm^−1^ are present [42], while the broad intense band at 3307 cm^−1^ is assigned to the stretching of the OH groups. The position and shape of this band mainly depends on the presence of weak-bound water that is also confirmed by the band at 1635 cm^−1^ [21]. These signals suggest that the treatment at 60 °C does not completely remove the water bound to the materials during their syntheses. The signals between 1000–400 cm^−1^ are due to the bending vibrations of Ti–OH and Ti–O, O–Ti–O bonds [17,42]. As expected, the spectra of the samples after the thermal treatment at different temperatures higher than 60 °C (curves b–e) confirmed the absence of water or of any organic substance: only the broad intense band at 600 cm^−1^ attributed to the Ti–O and O–Ti–O groups is visible [17]. Furthermore, by comparing these spectra it is possible to observe that the band at 600 cm^−1^ changes shape and intensity, due to the presence of different crystalline phases, as shown by XRD spectra elsewhere [43].

#### 3.2.3. SEM Analysis

**Figure 6:**
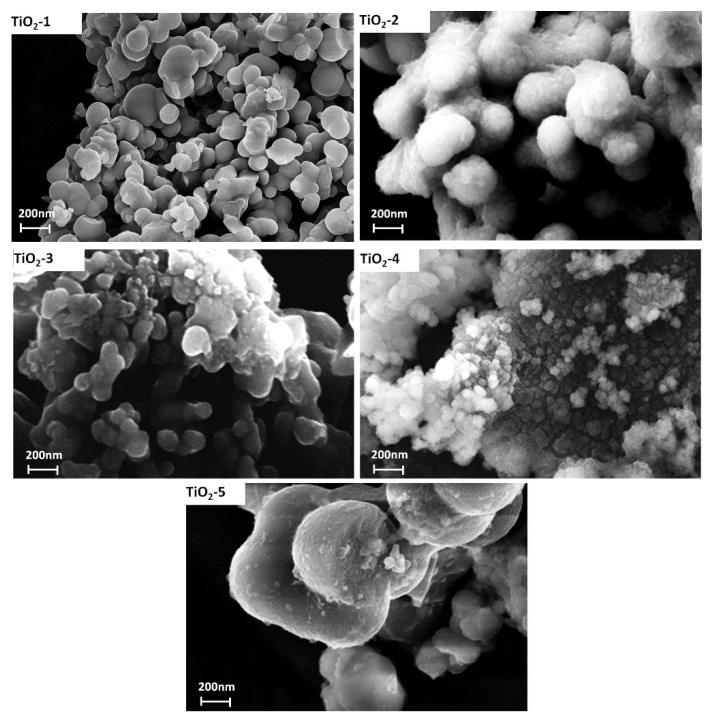
SEM micrograph of all samples.

The SEM micrographs of the TiO_2_ samples treated at different temperatures are shown in Figure 6. The images indicate that the particles have non-uniform size with a high degree of agglomeration. The nanoparticles size of the amorphous TiO_2_-1 sample is about 700 nm and no aggregate particles are clearly visible, due to the presence of both water molecules and hydroxyl groups that avoid the aggregation among particles [44]. When the temperature of the thermal treatment increases from 25 to 400 °C and from 25 to 600 °C at a heating rate of 9 °C/min (TiO_2_-2 and TiO_2_-3, respectively) or when the wet precipitates are kept at 600 °C for 1 h (TiO_2_-5 sample), then a sintering process occurs. In fact, it is known that calcination usually led to sintering of nanoparticles [19], although it is possible to find particles having a nanometric size.

On the other hand, the SEM images of TiO_2_-4 (heated at 60 °C and then placed in a hot muffle at 600 °C) shows particle size smaller than the others (~100 nm), accompanied by a high degree of agglomeration. The thermal treatment affected the crystal growth, indicating aggregation of TiO_2_ nanoparticles when the temperature increases. As a result of this treatment a mixture of crystalline phases (anatase and rutile) at different percentages depending on the different conditions adopted is found [45] (Table 2), as shown also by XRD analysis. In particular, comparing the results of XRD and SEM, a high percentage of rutile lead to crystal growth [39]. This effect has been highlighted in the TiO_2_-4 sample (characterized by the smaller particles size), which contains 85% of anatase and 15% of rutile (Table 2). When the transformation of anatase in rutile phase occurs, the nanoparticles size increase, because the transformation from anatase to rutile is due to a nucleation and growth process, in fact, the transition to rutile is accompanied by significant grain growth, resulting in large rutile grains and small anatase grains [46].

The obtained results are in agreement with those reported in literature [47,48]. Indeed, Sasani Ghamsari et al. [49] synthesized TiO_2_ nanoparticles by the sol–gel method and obtained very small particle even if the presence of agglomeration among the particles is evident. They showed that the particle size increases by calcinations and aging. Instead, Mahshida et al. [50] concluded that the rate of particle growth and the final particle size were a function of molar ratios. The crystallite size of the powder with lower molar ratio values tends to grow more rapidly when heated up to higher temperatures. The final size of the crystallites is determined by the relative rate of particle growth. Increase of temperature not only accelerates nucleation rate but also enhances particle growth. Furthermore, also, the synthesis procedure affects the nanoparticles size. The synthesis of TiO_2_ nanoparticles with controlled sizes by many methods was extensively studied [21]. Using microemulsion-mediated method, TiO_2_ nanoparticles with a size of 10–20 nm range were synthesized, but a strong agglomeration was observed [51]. Zhanget al. [52] synthesized amorphous TiO_2_ by hydrolysis of titanium ethoxide at the ice point obtaining TiO_2_ aggregates nanoparticles with a size of 0.2 µm. Vijayalakshmi et al. [53] have compared two different main methods for the synthesis of nanoparticles: the sol–gel route and hydrothermal route. They concluded that the TiO_2_ nanoparticles prepared via the sol–gel route are highly crystalline and have smaller crystallite size (~7 nm) than to the one prepared by the hydrothermal method (~17 nm). Comparing the different synthesis techniques, the sol-gel method is the most suitable method to prepare small nanoparticles, because it is possible to adapt the operative conditions of the synthesis, which play an important role on the size of the resulted nanoparticles [23].

## 4. Conclusions

In the present study TiO_2_ nanoparticles were synthesized by sol-gel method. After the synthesis, the nanoparticles were heated at different temperatures. FTIR and XRD analyses suggest that an increase of the calcination temperature induces the formation of a mixed-phase crystalline system with variable percentages of anatase and rutile. The results obtained by SEM suggest that an increase in the particles size accompanied by a noticeable aggregation of TiO_2_ nanoparticles is due to the achievement of high temperatures of the thermal treatments either and the presence of different content of the two crystalline phases of titanium dioxide. In conclusion, comparing the obtained results, it is possible to hypothesize that the high percentages of rutile negatively affect the nanoparticles size. Thus, the future goal will be to reduce the agglomeration and to preserve the nano size of the particles by a fine control of water–precursor molar ratio and the treatment temperature.

## Figures and Tables

**Table 1:** Different heat treatments of the TiO_2_ nanoparticles.

Samples	Heat Treatments
TiO_2_-1	60 °C-72 h
TiO_2_-2	25 °C–400 °C-2 h at 9 °C/min
TiO_2_-3	25 °C–600 °C-2 h at 9 °C/min
TiO_2_-4	600 °C-1 h
TiO_2_-5	wet precipitates at 600 °C-1 h

**Table 2:** Different percentages of anatase and rutile content depending on the different heat treatments of the TiO_2_ nanoparticles.

Samples	Heat TREATMENTS	Anatase %	Rutile %
TiO_2_-1	*T* = 60 °C for 72 h	-	-
TiO_2_-2	From 25 to 400 °C at 9 °C/min + *T* = 400 °C for 2 h	100	-
TiO_2_-3	From 25 to 600 °C at 9 °C/min + *T* = 600 °C for 2 h	30	70
TiO_2_-4	*T* = 600 °C for 1 h	85	15
TiO_2_-5	wet precipitates *T* = 600 °C for 1 h	68	32
